# Contribution of sustained attention abilities to real-world academic skills in children

**DOI:** 10.1038/s41598-023-29427-w

**Published:** 2023-02-15

**Authors:** Courtney L. Gallen, Simon Schaerlaeken, Jessica W. Younger, Project iLEAD Consortium, Jessica Wise Younger, Kristine D. O’Laughlin, Joaquin A. Anguera, Silvia A. Bunge, Emilio E. Ferrer, Fumiko Hoeft, Bruce D. McCandliss, Jyoti Mishra, Miriam Rosenberg-Lee, Adam Gazzaley, Melina R. Uncapher, Joaquin A. Anguera, Adam Gazzaley

**Affiliations:** 1 Department of Neurology, University of California San Francisco, San Francisco, CA 94158 USA; 2 Neuroscape, University of California San Francisco, San Francisco, CA 94158 USA; 3 Department of Psychiatry, University of California San Francisco, San Francisco, CA 94158 USA; 4 Department of Physiology, University of California San Francisco, San Francisco, CA 94158 USA; 5 Department of Psychology & Helen Wills Neuroscience Institute, University of California, Berkeley, Berkeley, USA; 6 Department of Psychology, University of California Davis, Davis, USA; 7 Department of Psychological Sciences and Brain Imaging Research Center (BIRC), University of Connecticut, Storrs, USA; 8 Graduate School of Education, Stanford University, Stanford, USA; 9 Department of Psychiatry, University of California San Diego, La Jolla, USA; 10 Neural Engineering and Translation Labs, University of California San Diego, La Jolla, USA; 11 Department of Psychology, Rutgers University, Newark, USA; 12 Advanced Education Research and Development Fund, Oakland, USA

**Keywords:** Human behaviour, Attention

## Abstract

Sustained attention is a critical cognitive ability that improves over the course of development and predicts important real-world outcomes, such as academic achievement. However, the majority of work demonstrating links between sustained attention and academic skills has been conducted in lab-based settings that lack the ecological validity of a more naturalistic environment, like school. Further, most studies focus on targeted academic measures of specific sub-skills and have not fully examined whether this relationship generalizes to broad measures of academic achievement that are used for important, real-world, academic advancement decisions, such as standardized test scores. To address this gap, we examined the role of sustained attention in predicting targeted and broad assessments of academic abilities, where all skills were assessed in group-based environments in schools. In a sample of over 700 students aged 9–14, we showed that attention was positively related to performance on targeted assessments (math fluency and reading comprehension), as well as broad academic measures (statewide standardized test scores). Moreover, we found that attention was more predictive of targeted math sub-skills compared to assessments of broad math abilities, but was equally predictive of reading for both types of measures. Our findings add to our understanding of how sustained attention is linked to academic skills assessed in more ‘real-world’, naturalistic school environments and have important implications for designing tools to support student’s academic success.

## Introduction

Sustained attention is a key cognitive ability that enables individuals to sustain their focus, inhibit impulses, and ignore distractions, and improves markedly over development^1–5^. Attention is one of several core executive functions (EFs) that are critical to successful goal-directed behavior^6–8^ and, importantly, are linked with real-world functional outcomes, such as academic achievement and mental and physical health^9–11^. Historically, researchers have measured sustained attention and academic abilities using targeted, objective assessments administered in highly controlled settings, such as a traditional research lab. This approach allows for significant experimental precision, where the procedures can be standardized and specific sub-skills of broader academic abilities (e.g., reading fluency) can be examined. Over the last decade, work using this methodology has demonstrated that sustained attention is linked to academic performance in such ‘lab-based’ environments, specifically when using direct assessments, or objective cognitive tasks, to quantify attention abilities^9,12–17^. Indeed, behavioral models suggest that stronger sustained attention abilities support skills important for both math (e.g., planning and carrying out a solution to a math problem) and reading (e.g., reading more words and reading more accurately)^18,19^.

These lab-based studies using direct assessment tools provide important information regarding the mechanisms by which sustained attention can influence academic abilities. However, it is critical to test how these mechanisms manifest in more ‘real-world’, or ecologically valid, contexts for students, such as classroom and other school settings, to capture the full breadth in which these skills are practiced, applied, and evaluated daily^20^. Although there are an increasing number of school-based studies that use cognitive tasks to measure EF abilities, they typically mimic controlled lab-based settings, where a researcher individually assesses a student outside the classroom in a quiet area such as a library^21–25^. These individualized assessments minimize distractions typical to school but, as such, do not assess student’s abilities in naturalistic environments with high ecological validity^20,26–28^.

One other common approach to assess children’s EFs is adult reports, where parents, teachers, or other caregivers rate a child’s behaviors using standardized questionnaires. While these methods can capture more complex and multi-component behaviors employed in real-world environments, they have several known limitations compared to direct assessments (i.e., cognitive tasks)^20,26–28^. First, adult reports are limited in their conceptual precision. They tend to survey broad behaviors and therefore often cannot isolate specific EF sub-components, such as sustained attention. Further, they often focus on behavioral challenges or children who are struggling (sometimes with a clinical focus). Therefore, these methods are less sensitive at capturing the full distribution of abilities and small skill differences between students, especially in typically developing children. Second, adult reports are quite limited in their objectivity. Adults may over-rate positive behaviors due to social desirability bias and their responses can even be affected by their own personality factors and stress levels. Moreover, adults may find it difficult to disentangle specific EF abilities from other information about the child such as their academic skills or overall temperament.

Direct assessments, especially digital cognitive tasks, address important limitations surrounding measurement precision and objectivity^20,26–28^, which are both critical when understanding individual differences in specific EFs such as sustained attention. However, there is clearly a need to further develop these methods to improve their ecological validity. One such approach is to directly assess EFs in group-based school settings rather than the typical *individualized assessments* conducted in a research lab or in school. Children spend a significant amount of time in school, and it is therefore essential to study their abilities in naturalistic settings to further our understanding of the classroom and more broadly, school, as a context for development^20,26,27,29^. Limited work in this area has begun to develop new methods for group-based EF assessments using digital cognitive tasks^20^. In this study, students completed EF tasks assessing inhibitory control, cognitive flexibility, and working memory on tablets in naturalistic classroom settings with their peers. Performance on the group-based assessments of EFs were related to performance on the same tasks administered individually (outside the classroom, in a quiet space in the school). Both the group-based and individual assessments predicted teacher ratings of self-regulation behaviors in the classroom and students’ statewide standardized test scores. Importantly, though, only performance on the group-based tasks were related to measures of academic growth (two-year improvement on standardized test scores), suggesting that assessments conducted in more naturalistic contexts provide unique insights into how cognitive skills are linked to academic performance^20^.

In addition to more naturalistic assessment environments, it is also important to consider the generalizability of the assessments themselves. In mechanistic studies, academic abilities, such as math and reading, are often measured with targeted tests of specific sub-skills, such as tests from the Woodcock-Johnson Tests of Achievement^30^ or the Wechsler Individual Achievement Test^31^. Yet, in school, students must integrate multiple sub-skills, which may be better captured by more broad measures that comprehensively assess academic performance, such as grades or standardized test scores. Further, educators and parents often focus on results of these broad types of tests to evaluate scholastic achievement, and it is these results that are used for important academic advancement decisions, such as college readiness. However, few studies have examined the direct contribution of sustained attention to such measures (e.g., grades^32,33^) and, moreover, whether the contributions of attention to targeted (e.g., Woodcock-Johnson) and broad (e.g., standardized test scores) academic assessments may differ. A more comprehensive understanding of the relationship between sustained attention and academic abilities can help researchers and educators develop methods to better support students in real-world contexts, such as school^34,35^. As such, examinations of how sustained attention supports performance in various academic areas is critical for making a meaningful impact in our students.

To this end, we leveraged a rich dataset that assessed sustained attention and both targeted and broad measures of academic abilities in over 700 students in 4th through 8th grade (a sub-sample of^36,37^. Importantly, all measures were assessed in more naturalistic, group environments in school (compared to individualized assessments), allowing us to evaluate sustained attention and academic skills in contexts with significant real-world interference and distraction, much like students' typical learning environments. First, we examined whether attention was related to targeted tests of math and reading sub-skills (math fluency and reading comprehension), when assessed in school contexts. Second, we asked whether attention would similarly predict broad measures of academic abilities (statewide academic achievement tests of math and English Language Arts). Finally, we compared the predictive power of attention between academic subjects (i.e., math and reading) and types of academic tests (i.e., targeted and broad) to assess whether attention was more strongly linked to a particular subject or type of test. In doing so, we aim to more comprehensively understand how sustained attention contributes to academic performance in real-world, naturalistic school environments.

## Methods

This study used a subset of data from Project iLEAD. The data collection methodology is described in detail in the original Project iLEAD report^36,37^ and summarized below.

### Participants

The entire iLEAD dataset includes responses from 1280 students (female, n = 630) from nine schools in Northern California (7 public, 1 private, 1 parochial), and was collected at four time points over a two-year period (two time points each year, once in the Fall and once in the Spring). In this study, we focused on data collected from the final time point of data collection (Spring of year 2), collected when students were in grade 4, 6, or 8 (n = 983, 500 female). We then restricted our sample to those students who used the same standardized testing procedures, excluding students classified by the district as English Language Learners (n = 126) and those with a special education diagnosis (n = 108, 32 of which were also excluded for being English Language Learners). We further excluded students who did not complete the cognitive tasks at this timepoint (sustained attention and/or basic response time) or were removed from the standard RT-based outlier cleaning methods as performed in the original iLEAD work^36,37^ (n = 56; see below for more details on cognitive tasks and outlier cleaning). Specifically, 38 students (4.9%) did not complete the cognitive tasks due to technical issues on the day of assessment (N = 2 for sustained attention; N = 36 for basic response time) and 18 (2.3%) were removed as RT-based outliers (N = 16 for sustained attention; N = 2 for both sustained attention and basic response time). The final subset of the data analyzed in this study therefore included 725 unique students (n = 360 females), although not all students completed both the targeted and broad academic assessments, with some completing only one type of assessment and a smaller subset completing both. Students were diverse in such demographic characteristics such as parental level of education, ethnicity, language fluency, and income level (Table 1).

**Table 1:** Sample characteristics (Spring Year 2).

		N = 725
Age (years)	Mean (stdev); range	12.4 years (1.6); 9.5–14.9
Gender	Females (F)	360
	Males (M)	365
Grade	4	181
	6	218
	8	326
Ethnicity	Asian	266
	Hispanic	163
	Other	96
	White	127
	Not available	73
Language fluency	English	321
	IFEP	57
	RFEP	274
	Not available	73
Low income	No	465
	Yes	187
	Not available	73
Parent education	Not HS graduate	35
	HS graduate	75
	Some college	85
	College graduate	208
	Grad school or post-grad	235
	Not available	94
Assessment Availability	Targeted academic tests	710
	Broad academic tests	708
	Both targeted and broad academic tests	598

Certain characteristics were not available for some students, which we report as ‘Not available’. *F* Female, *M* male, *IFEP* initially fluent english proficient, *RFEP* reclassified to fluent english proficient, *HS* high school.

We conducted this study according to protocols approved by the Institutional Review Board (IRB) of the University of California San Francisco. All methods were carried out in accordance with relevant guidelines and regulations for experimental protocols approved under UCSF IRB #13-10917. We obtained written informed consent from the parents or guardians of all participants at the beginning of the study and verbal assent from all participants before all data collection sessions. At the end of the study, all students (regardless of participation) received snacks and stickers.

### Procedure

At each of the four timepoints, digital EF assessments were administered first, and the research team returned several weeks later to administer digital, targeted tests of math and reading abilities (M = 5.7 weeks, SD = 2.4, min. = 1.9, max. = 10). All digital assessments were completed on iPads and were collected in a variety of school environments, including more traditional classroom contexts (e.g., a teacher’s 3rd grade class or a teacher’s science class) as well as libraries, cafeterias, and gymnasiums. Critically, sessions were administered in group settings during school (including traditional ‘classroom’ environments) and 85% (34/40) of the data collection sessions at this timepoint were conducted within a single grade. Further, even in the case of non-traditional classroom environments, all students in assessment groups shared at least one ‘class’ together (e.g., students may have shared a gym class, but otherwise did not share classes). Collectively, these assessment environments can be considered more ecologically valid, group-based school contexts compared to prior work in lab-based settings or one-on-one assessments in a quiet school room such as a library.

Sessions were facilitated by a group of trained researchers (4–12 researchers depending on student group size, which ranged from 7 to 83 students (mean = 33 students)). A range of group size for assessment sessions was necessary for some schools with more students (e.g., to collect assessment data in a more time-efficient fashion). Importantly, sessions with more students typically had more research facilitators present and potential differences in effects associated with school size should be accounted for by including a random effect of school in our linear mixed models (see below). Researchers monitored participants, ensured that they understood the tasks, provided additional technical assistance as needed, and answered student questions. A school staff member and/or Neuroscape researcher were present at all data collection sessions. These sessions lasted approximately 50 minutes.

At the end of the study, the school district provided retrospective academic achievement measures for participants whose parents consented to the sharing of district data. District data included the following: (1) demographic data, such as that included in Table 1; (2) academic data for each year of data collection, including statewide standardized tests [Smarter Balanced Assessment Consortium (SBAC) standardized test scores for math and English Language Arts (ELA)] and grades for science, math, and ELA; (3) special education diagnosis/Individual Education Plan categorization; and (4) other data such as attendance records and number of disciplinary incidents.

### Digital sustained attention and other executive function (EF) assessments

To rapidly assess a variety of EFs in group settings, the iLEAD project utilized a novel digital assessment battery, Adaptive Cognitive Evaluation Classroom (ACE-C). Each task was developed from cognitive assessments commonly used in lab-based settings and modified to include adaptive algorithms, motivational trial-by-trial and end-of-task feedback, and a user-friendly interface. The adaptive algorithms used a psychometric staircase approach, which allowed students to perform the same tasks over multiple assessment time points without being confounded by floor or ceiling effects. Moreover, the same task could measure an individual’s changing cognitive abilities over time and be compared with students of different ages, genders, races, and cultures^36–38^. The battery included 9 tasks designed to measure aspects of EF such as attention, working memory, and multitasking. In addition, an ACE-C measure of basic response time (BRT) was included at the beginning of each session. The order of ACE-C tasks was kept consistent across sessions.

Here, we used ACE-C data from one task designed to assess sustained attention abilities, the ACE-C continuous performance task (CPT; for full details on the ACE-C battery see Refs.^36,37^). Sustained attention abilities are commonly assessed with CPTs, where participants maintain their focus over time to respond to target stimuli and inhibit responses to non-target stimuli^39^. The ACE-C CPT was adapted from the Test of Variables of Attention (TOVA)^40^, in which participants viewed a symbol that appeared either at the top or bottom of the iPad screen. They were instructed to press a button with the index finger on their dominant hand when the symbol appeared at the top of the screen (target) and to withhold responses when it appeared at the bottom of the screen (non-targets). The task had two conditions that varied in their proportion of target stimuli to measure different aspects of sustained attention commonly assessed in CPTs. As in TOVA, the first condition (‘sustained’) had infrequent targets that occurred for 33.3% of trials and the second condition (‘impulsive’) had frequent targets that occurred for 66.6% of trials. Participants completed 10 practice trials (six target, four non-target trials), then 80 experimental trials (40 in each condition), where conditions were completed in a fixed order (sustained before impulsive). We computed 3 CPT performance metrics, separately for each condition: (1) response time (RT) for correct target trials, (2) response time variability (RTV) for correct target trials, and (3) accuracy comparing correct responses to target stimuli (‘hits’) and incorrect responses to non-target stimuli (‘false alarms’) as d-prime (d’).

For both BRT and CPT, data cleaning was performed in accordance with the original iLEAD work^36,37^. We first excluded trials with no responses when a response was expected and excluded anticipatory trials (RT < 200 ms). Further, individual students were excluded from analyses using RT-based outlier criteria. Within each grade, students were defined as outliers if their performance fell outside three median absolute deviations (MADs) of the median performance for their cohort (grade).

### Targeted academic assessments

Targeted tests of academic skills were examined using digital assessments of math, reading, and reasoning skills. These tasks were modified from those commonly used in lab settings and were adapted for group administration in schools. Here, we focused on two tasks related to academic sub-skills in this assessment battery, that were based on standardized measures of math and reading: math fluency and reading comprehension. We did not examine any other, more experimental, tasks developed for this battery. For both assessments, stimuli were presented on the iPad screen until a response was made. Participants were asked to answer as many questions as possible in three minutes and two practice trials were conducted before each task began to ensure comprehension.

Math ability was measured using an iPad task similar to the Math Fluency task of the Woodcock-Johnson III Tests of Achievement^30^. This task required participants to look at single-digit math equations (addition, subtraction, and multiplication) and type in the correct answer. The math fluency score was calculated as the total number of correct answers in the three-minute period.

Reading ability was assessed using stimuli from the Test of Silent Reading Efficiency and Comprehension (TOSREC^41^) presented on an iPad. This task required participants to read and comprehend grade-appropriate sentences (e.g., “It can be cold in the winter”), by indicating if they were ‘True’ or ‘False’ with a button press on the iPad. The reading comprehension score was calculated as the number of correct answers minus the number of incorrect answers in the three-minute period.

### Broad academic assessments

As described above, the school district also shared results from statewide standardized academic testing. Here, we used the Smarter Balanced Assessment Consortium (SBAC) scores for math and English Language Arts (ELA) corresponding to the second year of data collection. SBAC scores were used as a metric of broad academic outcomes that more comprehensively assessed multiple sub-skills and required integration across sub-skills. Briefly, depending on student grade, the SBAC math test may have included sub-tests involving complex expressions, geometry, algebra, number comparisons (e.g., fractions or decimals), and other aspects of complex mathematical reasoning, while the SBAC ELA test may have included sub-tests involving passage and listening comprehension, vocabulary and synonyms, and grammar and punctuation. We did not use grades as a similar broad measure of academic achievement because they were not available for 4th grade students.

SBAC tests were administered by the school district in the classroom during the second part of the school year, around the same time of the iPad-based spring assessments. These scores were only available for students attending public schools and, further, those whose parents additionally consented for the school to share these data. Note that for simplicity, we sometimes refer to the SBAC ELA score as ‘broad reading’, although this test assessed some abilities beyond reading (e.g., listening comprehension).

### Analysis methods

To ensure our examination of the relationship between sustained attention and academic achievement reflected the specific contribution of attentional skill and not other related processes, we performed several transformations to the data. First, we regressed response times from the ACE-C Basic Response Time (BRT) task from each of the three CPT performance metrics (RT, RTV, d′), to remove general effects related to basic processing speed as well as motoric speed while using tablet-based technology to complete digital assessments, as we have done in our prior work with this dataset^36,37^. Then, we z-scored all attention and academic measures by grade to remove expected developmental effects on these metrics.

**Figure 1:**
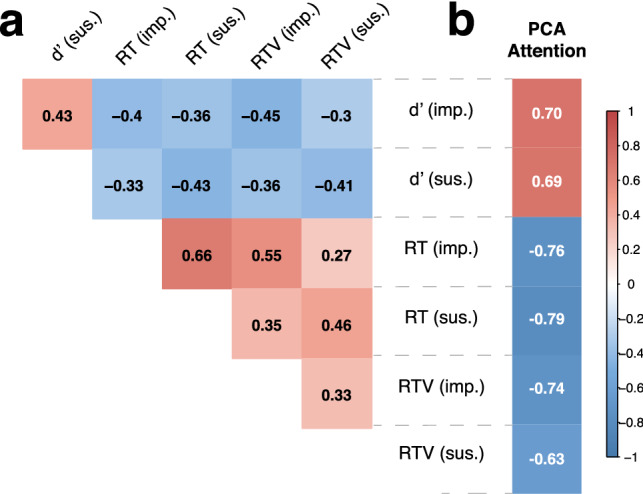
(**a**) Correlation map of the 6 CPT performance metrics. *RT* response time, *RTV* response time variability. (**b**) Loading scores of the principal component of sustained attention using EFA. *Sus* sustained condition, *imp* impulsive condition.

Next, we performed a data reduction technique, Principal Component Analysis (PCA), on the 6 CPT performance metrics, given that they were highly related (Fig. 1a) and to reduce the number of attention predictors (and therefore the total number of regression analyses performed) (Supplementary information text 1). Using PCA with the "psych" package in R^42^, we found one principal component represented more than half of the total variance (Table S1). We used the estimated component score from this single component as our main sustained attention variable throughout our analyses, where all 6 performance metrics contributed to this component. Accuracy (d′) loaded positively onto this attention variable while RT and RTV loaded negatively, indicating the variable reflects higher accuracy and faster, more consistent RTs on both the sustained and impulsive conditions of the ACE-C CPT (see Fig. 1b for PCA loading scores for each performance metric).

To examine the relationships between this sustained attention variable and academic skills, we computed linear mixed models (LMMs) using package "lmer" in R^43^ for each assessment type (targeted and broad) and subject (math and reading). Random effects included school to reflect the clustering of students within schools. The fixed effects included the estimated component score of sustained attention and confounding demographic variables (language proficiency, parental education level, ethnicity, and gender). We included these demographic variables to assess the unique proportion of variance specifically explained by sustained attention, as demographic variables have also been shown to have a strong relationship with academic achievement^44–49^. We did not include age or grade in the models as all attention and academic measures were z-scored by grade in a prior processing step. Further, we included parental education level, and not low-income status (Table 1), as a proxy for student socioeconomic status in the model, as these variables are highly related and parental education level provides a more continuous measure of this demographic characteristic.

We computed F-tests for main effects to determine the impact of sustained attention on academic performance and for interaction effects to determine whether assessment type (targeted or broad) or academic subject (math or reading) had a differential influence on the relationship with academic performance. We report effect sizes in accordance with the approach of Nakagawa and Schielzeth implemented in the R package "MuMIn"^50^. Specifically, we report R2m, the variance explained by the fixed factors (without random effects), and R2c, the variance explained by the entire model (both fixed and random effects) following suggested guidelines: very weak = 0 to < 0.02; weak = 0.02 to < 0.13; moderate = 0.13 to < 0.26; substantial ≥ 0.26^51^. Note that, although these guidelines are based on linear regression rather than linear mixed models, the effect sizes are comparable between models. We calculated global effect sizes for each statistical model. All p-values within each model were false discovery rate (FDR) corrected. We set a significance threshold of p < 0.05, but we report non-significant 'trends' at p < 0.1.

### Ethics approval statement

We conducted this study according to protocols approved by the Institutional Review Board (IRB) of the University of California San Francisco (IRB #13-10917). We obtained written informed consent from the parents or guardians of all participants at the beginning of the study and verbal assent from all participants before all data collection sessions.

## Results

### Predicting performance on targeted academic tests from sustained attention abilities

**Figure 2:**
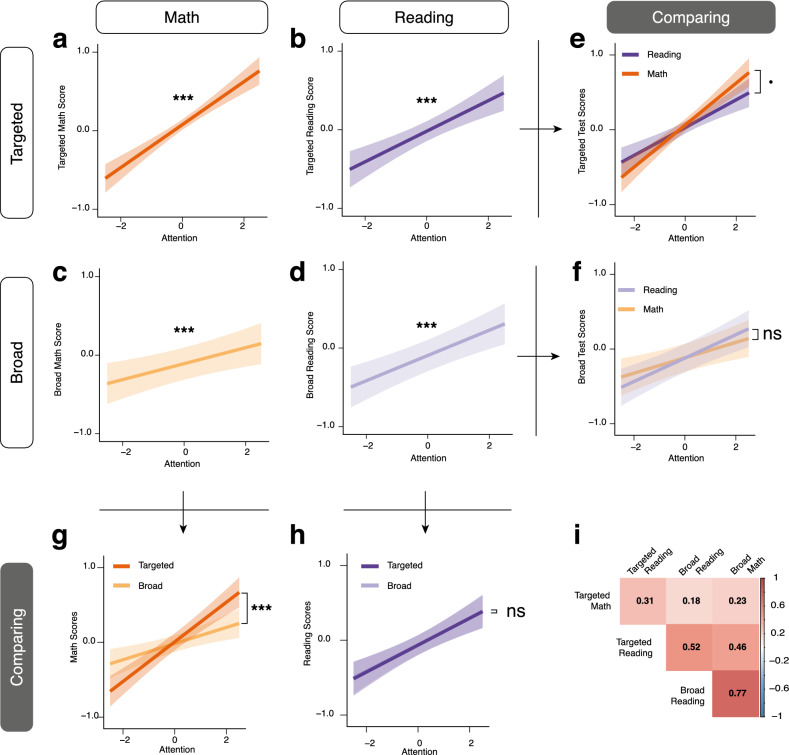
Predicted main effect of sustained attention on targeted tests assessing (**a**) math and (**b**) reading, and on broad tests of (**c**) math and (**d**) ELA/reading. Interaction effect of sustained attention between math and reading in (**e**) targeted measures and (**f**) broad measures. Interaction effect of sustained attention between targeted and broad measures in (**g**) math and (**h**) reading. (**i**) Correlation matrix of all math and reading test scores. [*ns* not-significant, •p < 0.1, *p < 0.05, ***p < 0.001].

Linear mixed models revealed the sustained attention component score showed a significant, unique contribution to performance on tests of targeted academic abilities, after accounting for BRT (regressed prior to PCA to create the attention factor) and demographic factors (included in the linear mixed model; Fig. 2a,b). Specifically, attention showed positive main effects on math fluency scores (F(1, 571) = 65.60, p < 0.001, R^2^_m (model)_ = 0.27, R^2^_c (model)_ = 0.27) and reading comprehension scores (F(1, 569) = 25.30, p < 0.001, R^2^_m (model)_ = 0.13, R^2^_c (model)_ = 0.14) (Table S2). We then directly compared the differential predictive power of attention on reading and math skill in a new model with an academic subject interaction term. We found that attention had a stronger predictive effect on the targeted test of math than on reading at a ‘trend’ level (F(1, 1153) = 3.50, p = 0.06, R^2^_m (model)_ = 0.19, R^2^_c (model)_ = 0.19) (Fig. 2-e).

### Predicting performance on broad academic tests from sustained attention abilities

Using similar linear mixed models, we next tested whether sustained attention had a similar predictive effect on performance on tests of broad academic abilities (SBAC math and ELA test scores). Similar to the targeted measures (Fig. 2c,d, Table S2), attention showed positive main effects on measures of math (F(1, 621) = 9.65, p = 0.002, R^2^_m (model)_ = 0.29, R^2^_c (model)_ = 0.35) and ELA (F(1, 621) = 22.70, p < 0.001, R^2^_m (model)_ = 0.25, R^2^_c (model)_ = 0.30). We again directly compared the predictive power of attention between academic subjects (Fig. 2f). Interestingly, unlike the targeted measures, we did not find a significant interaction between attention and academic subject, suggesting that attention did not differentially predict broad math and reading (SBAC ELA) test scores (F(1, 1260) = 1.30, p = 0.24, R^2^_m (model)_ = 0.27, R^2^_c (model)_ = 0.32) (Fig. 2f).

### Comparing sustained attention relationships with targeted and broad academic measures

Given the differing results of whether sustained attention more strongly predicts math and reading on targeted and broad measures, we next directly compared the attention performance predictions for each type of measure, allowing us to test whether sustained attention abilities are more predictive of one type of measure (i.e., targeted or broad) for math and reading. We first examined relationships between the four measures (targeted and broad math and reading) and found that there were significant relationships among them, albeit with very different magnitudes (Fig. 2i).

We then confirmed that there was an interaction between assessment type (targeted, broad) and subject (math, ELA/reading) on predicting attention (F(1, 2426) = 4.71, p = 0.03, R^2^_m (model)_ = 0.22, R^2^_c (model)_ = 0.24) When comparing the two types of academic assessments for math, we found that attention was more predictive of targeted than broad math scores (F(1, 1207) = 11.29, p < 0.001, R^2^_m (model)_ = 0.28, R^2^_c (model)_ = 0.29; Fig. 2g, Table S3). However, when comparing the two types of academic assessments for reading, we found that attention was equally predictive of both types of reading measures (F(1, 1209) = 0.004, p = 0.94, R^2^_m (model)_ = 0.19, R^2^_c (model)_ = 0.20; Fig. 2h, Table S3).

## Discussion

The findings of this study demonstrate that sustained attention abilities are predictive of academic skills assessed in group-based, more naturalistic, school settings compared to prior work using individualized assessments. Importantly, we found links between attention and academic skills both for targeted measures that assess specific academic sub-skills and broad measures that more comprehensively assess and require integration across academic skills. Using a large dataset of children, we first identified a composite sustained attention metric derived from a digital continuous performance task (CPT). We then examined the unique contribution of sustained attention to academic abilities, specifically focusing on reading and math, while controlling for influences from demographics and performance on a basic response time task that controls for differences in processing speed using tablet-based digital measures. We extended findings of previous work, observing strong relationships between attention and targeted reading and math measures, critically showing that these patterns also exist when they are assessed in more ‘real-world’, group-based school contexts with higher ecological validity. We next showed similar relationships with standardized ELA/reading and math tests used for the broad assessment of academic abilities. Finally, we compared the attention metric prediction between academic subjects and types of tests, which revealed new evidence of the types of academic measures that sustained attention abilities may most strongly support, particularly in naturalistic school settings. Below we discuss the implications of these results and potential directions for future research.

First focusing on the targeted measures, we observed strong positive relationships between math and reading performance and sustained attention ability, corroborating a significant body of previous work^12–15,21–24^. The present findings importantly expand on this previous literature by using digital versions of these tests delivered in group-based, school settings. These more naturalistic school environments placed significant demands on students’ abilities to stay on task and monitor their performance in the presence of ongoing distractions^26^ and therefore have significantly stronger ecologically validity than prior research that assessed similar skills in highly controlled lab-based contexts or one-on-one assessments in school. Thus, our results reveal that these relationships remain present even when the testing is performed in more ecologically valid environments outside the lab, advancing our understanding of the relationship between attention and academic abilities in naturalistic contexts that are critical for students’ real-world success.

We then extended these analyses to broad measures of academic achievement that more comprehensively assessed and required integration across academic sub-skills (statewide academic achievement scores). We notably found similar patterns: sustained attention was positively related to broad tests of math and reading/ELA abilities. These results are in line with the relatively few reports showing attention is related to broad academic measures, such as grades^32,33^, although these studies did not additionally examine targeted measures of academic sub-skills. The statewide standardized tests examined here involved integrating skills from multiple domains, such as complex problem solving, mathematical reasoning, and paragraph and listening comprehension. For math, students with stronger attentional control may better suppress irrelevant information and immature strategies to aid in solving more complex math problems^52,53^. For reading, stronger attention skills may support more advanced reading abilities^54^, by helping readers focus on relevant information when reading longer text and providing resources for understanding phonemic structure to decode new words^55^. Better understanding the relationship between sustained attention and broad measures of academic performance provides a foundation for taking a student’s cognitive profile into account (e.g., strengths in sustained attention relative to other EFs) when predicting and aiming to improve their academic performance.

Finally, we provided new evidence for how sustained attention differentially predicts performance for academic subjects (math and ELA/reading) and types of tests (targeted and broad). Although attention was related to all four academic measures independently, these direct comparisons offer insight into specific types of subjects and measures that sustained attention may more strongly support. First, comparing subjects, attention was more predictive of targeted math than reading (at a ‘trend’ level), but was equally predictive of broad math and reading. Similar results from a recent meta-analysis showed inhibitory control (a related EF) was more strongly associated with tests of math compared to reading abilities^56^. Second, comparing types of tests, attention was more predictive of targeted than broad math, but was equally predictive of both types of reading tests. Taken together, these results show that sustained attention may be most strongly predictive of the timed, targeted math assessment and, as such, perhaps solving relatively simple stimuli that benefit from memorization and retrieval. Although these abilities undoubtedly support completing more complex math problems, the relatively lower correlation between targeted and broad math scores suggests that there are additional skills tapped in the broad math assessment that may be supported by processes beyond sustained attention. For reading, although the targeted test involved reading simple sentences in a similarly speeded manner, it also required participants to make a true or false judgment about the sentence, therefore involving both fluency and comprehension abilities. Interestingly, sustained attention seems to equivalently contribute to these simpler reading abilities as well as the complex skills assessed in the broad ELA/reading assessment that required skills such as vocabulary and grammar as well as passage and listening comprehension. These results lay the groundwork for better understanding how sustained attention can differentially contribute to performance on several types of academic measures that assess both targeted sub-skills and broad abilities. Moreover, future work should further examine the specificity of the relationships between various EFs and academic abilities.

Although these results provide important insights into how sustained attention contributes to academic abilities, there are a few limitations that could be addressed in future work. First, given the correlational nature of our analyses, it is important to examine causal relationships between sustained attention and academic achievement. As real-world academic abilities are typically the focus of parents and educators when assessing scholastic success, future work could examine whether improving sustained attention (e.g., through a training intervention) would have downstream effects on real-world academic skills^57,58^. Second, our sustained attention measure was limited to a digital version of a continuous performance task (CPT) completed on an iPad. Future work could expand our mechanistic understanding of how attention is related to academic performance by examining moderating effects classroom behavior, other common assessments of sustained attention reported by educators and parents in surveys, and other components of attention abilities such as search or divided attention. Third, the population used for this study (children in ‘middle childhood’) is only a portion of the protracted development of sustained attention. Middle childhood is a particularly understudied period of development, and thus is important for broadening our understanding of how skills interactively emerge^59,60^, but is limited in understanding how sustained attention contributes to academic performance in later adolescence and beyond (e.g., college readiness). Future work could examine how attention abilities assessed at this age predict future academic success.

Finally, while ecological validity is not directly quantifiable, we hypothesize that our assessment contexts were more naturalistic compared to individually testing a student in a traditional research lab or outside the classroom in a quiet space in school. Our assessment environments sometimes included larger, mixed group settings (e.g., some contexts were mixed classes in a cafeteria or gymnasium) and were led by a research team, rather than exclusively focusing on traditional ‘classrooms’ and being led by a familiar teacher. Nonetheless, students were still assessed with their peers and, as such, these contexts placed significant demands on their abilities to perform tasks in the presence of ongoing distractions, much like typical in-school learning contexts. Further, these testing environments required students to employ other important skills from the classroom to succeed, such as directing their attention toward the researcher (much like they would a teacher), waiting for their turn to ask a question or start a task, and avoiding disrupting others while they completed their assignment. We acknowledge that supervision from less familiar researchers somewhat limits the ecological validity of the environment for this study. However, we hypothesize that our assessment contexts were nevertheless more naturalistic than prior individualized assessment work, both in lab and in school.

Future work related to this study could have a more direct focus on more traditional classroom contexts, where controlling for classroom-based factors (e.g., classroom size and noise or teachers’ instruction style) would be important to consider as well. Further, the assessments themselves could have stronger ecological validity. Direct assessments, such as the tablet-based tasks used here, are limited in their ability to assess more complex behaviors that integrate interpersonal dynamics or other social and emotional factors^20,27^. One less common method to assess EFs in children is naturalistic observation, where independent assessors rate children's behavior as they engage in an activity or task. Although this method has drawbacks related to experimental control, conceptual precision, and scalability (see Refs.^26,27^ for extended discussions), some new work has developed standardized methods for assessing EFs sub-components using interactive, group-based measures that incorporate situational tasks and classroom social dynamics^27^. More broadly, it is apparent that the different methods to measure EFs in students (direct assessments, adult reports, and observational tools) each have their own unique set of strength and weaknesses^26,28^. Future work that integrates measures of students’ EF performance across these approaches will further our understanding of how sustained attention and other cognitive abilities support academic performance.

The present study expands our understanding of how sustained attention abilities are linked to a variety of academic skills assessed in naturalistic school settings. Here, we provide support for previous work, showing that sustained attention is related to targeted metrics of math and reading sub-skills and add new evidence that this relationship extends to broad metrics of academic achievement. Importantly, by directly comparing metrics, we show that targeted math performance may be better predicted by sustained attention, but that attention is equally predictive of targeted and broad reading assessments. Collectively, this work adds to our growing understanding of how sustained attention is related to academic abilities, especially in naturalistic school contexts, and provides new avenues for developing attention interventions that could have benefits that extend to real-world academic success.

## Supplementary Information

Supplementary Information.

## Data Availability

Deidentified data will be stored at the Neuroscape Center at UCSF and will be made available upon reasonable request to one of the corresponding authors.
