# Supplementary material for: Contribution of sustained attention abilities to real-world academic skills in children
Source: Sci Rep. 2023 Feb 15;13:2673. doi: 10.1038/s41598-023-29427-w (PMC9932079; doi:10.1038/s41598-023-29427-w)
Supplement: Supplementary file 1 — Supplementary Information. [file 41598_2023_29427_MOESM1_ESM.pdf]

Contribution of sustained attention abilities to real-world academic skills in children

Supplementary Information Text 1

Courtney L. Gallen[1,2,#,*], Simon Schaerlaeken[1,2,#], Jessica W. Younger[1,2], Project iLEAD Consortium[2,5], Joaquin A. Anguera[1,2,3], Adam Gazzaley[1,2,3,4,*]

[1]Department of Neurology, University of California San Francisco, San Francisco, CA 94158

[2]Neuroscape, University of California San Francisco, San Francisco, CA 94158

[3]Department of Psychiatry, University of California San Francisco, San Francisco, CA 94158

[4]Department of Physiology, University of California San Francisco, San Francisco, CA 94158

[5]The Project iLead Consortium is composed of: Jessica Wise Younger, Kristine D. O'Laughlin, Joaquin A. Anguera, Silvia A. Bunge, Emilio E. Ferrer, Fumiko Hoeft, Bruce D. McCandliss, Jyoti Mishra, Miriam Rosenberg-Lee, Adam Gazzaley, and Melina R. Uncapher

[#]CLG and SS should be considered joint first authors

***Corresponding authors:**

Adam Gazzaley - adam.gazzaley@ucsf.edu

Courtney L. Gallen - courtney.gallen@ucsf.edu

Sandler Neurosciences Center

675 Nelson Rising Lane, Room 505

San Francisco, CA 94158

Creating a sustained attention variable

From the ACE CPT, 6 performance metrics were computed: d', reaction time (RT), and reaction time variability (RTV) for the sustained and impulsive CPT conditions. These 6 metrics were all highly correlated (Figure 1-a). To reduce the number of variables, we computed a Principal Component Analysis using the 6 metrics. We chose one component that represented 50% of the total variance. The part of the variance explained by each additional component is shown in the table below. The loadings of each variable onto this component are displayed in Figure 1-b. Accuracy (d’) loaded positively onto this principal component while RT and RTV loaded negatively, indicating that the component reflects higher accuracy, faster and less variable RT on the CPT. We used estimated component scores of this sustained attention variable in all subsequent models.

Table S1. Part of the variance explained by individual PC

Variance explained by each PC	
PC1	0.50
PC2	0.14
PC3	0.13

Table S2. Fixed-effects of the main linear mixed models assessing the relationship between sustained attention and math and reading while accounting for demographic factors.

Type III Analysis of Variance Table with Satterthwaite's method

Model	Fixed effect	df	F value	P value	Significance level
Targeted Math	Attention	1	53.402	<0.001	***
	Language.Fluency	2	0.1706	0.843	
	Gender	1	0.1215	0.728	
	Parent.Ed.Lvl	4	1.9623	0.099	.
	Ethnicity	3	34.1464	<0.001	***
Model	Fixed effect	df	F value	P value	Significance level
Targeted Reading	Attention	1	38.387	<0.001	***
	Language.Fluency	2	1.3739	0.254	
	Gender	1	6.1535	0.013	*
	Parent.Ed.Lvl	4	2.4214	0.047	*
	Ethnicity	3	9.4764	<0.001	***
Model	Fixed effect	df	F value	P value	Significance level
Broad Math	Attention	1	10.1485	0.002	**
	Language.Fluency	2	2.6761	0.070	.
	Gender	1	0.2943	0.588	
	Parent.Ed.Lvl	4	9.7223	<0.001	***
	Ethnicity	3	23.9359	<0.001	***
Model	Fixed effect	df	F value	P value	Significance level
Broad Reading	Attention	1	22.8925	<0.001	***
	Language.Fluency	2	0.1395	0.870	
	Gender	1	17.1693	<0.001	***
	Parent.Ed.Lvl	4	12.6896	<0.001	***
	Ethnicity	3	10.5632	<0.001	***

Table S3. Fixed-effects of the main linear mixed models assessing the relationship between sustained attention, its interaction with different conditions and test scores while accounting for demographic factors.

Type III Analysis of Variance Table with Satterthwaite's method

Model	Fixed effect	df	F value	P value	Significance level
Targeted	Attention	1	70.8853	<0.001	***
	Math/Reading	1	1.9697	0.161	
	Attention:Math/Reading	1	4.5746	0.033	*
	Language.Fluency	2	0.2468	0.781	
	Gender	1	3.2518	0.072	.
	Parent.Ed.Lvl	4	3.2635	0.012	*
	Ethnicity	3	27.0903	<0.001	***
Model	Fixed effect	df	F value	P value	Significance level
Broad	Attention	1	6.9362	0.009	***
	Math/Reading	1	0.1832	0.669	
	Attention:Math/Reading	1	4.5847	0.032	*
	Language.Fluency	2	1.014	0.363	
	Gender	1	4.6778	0.031	*
	Parent.Ed.Lvl	4	16.1718	<0.001	***
	Ethnicity	3	19.403	<0.001	***
Model	Fixed effect	df	F value	P value	Significance level
Math	Attention	1	38.8014	<0.001	***
	Targeted/Broad	1	1.9896	0.159	
	Attention:Targeted/Broad	1	22.249	<0.001	***
	Language.Fluency	2	1.279	0.279	
	Gender	1	0.0615	0.804	
	Parent.Ed.Lvl	4	9.7504	<0.001	***
	Ethnicity	3	39.8505	<0.001	***
Model	Fixed effect	df	F value	P value	Significance level
Reading	Attention	1	30.6846	<0.001	***
	Targeted/Broad	1	0.0438	0.834	
	Attention:Targeted/Broad	1	0.3329	0.564	
	Language.Fluency	2	0.4182	0.658	
	Gender	1	12.5647	<0.001	***
	Parent.Ed.Lvl	4	10.0867	<0.001	***
	Ethnicity	3	13.6179	<0.001	***
